# Epileptic Patient with Mild Malformation of Cortical Development with Oligodendroglial Hyperplasia and Epilepsy (MOGHE): A Case Report and Review of the Literature

**DOI:** 10.1155/2019/9130780

**Published:** 2019-06-09

**Authors:** A. Verentzioti, I. Blumcke, A. Alexoudi, P. Patrikelis, A. Siatouni, S. Korfias, D. Sakas, S. Gatzonis

**Affiliations:** ^1^Department of Neurosurgery, National & Kapodistrian University of Athens, Evangelismos Hospital, Athens, Greece; ^2^Department of Neuropathology, University Hospital Erlangen, Germany

## Abstract

**Introduction:**

There is an emerging interest in the literature about MOGHE (Mild Malformation of Cortical Development with Oligodendroglial Hyperplasia and Epilepsy). We report the case of an epileptic patient with MOGHE.

**Case Report:**

A 33-year-old male patient was suffering from refractory focal epilepsy since adolescence. MRI demonstrated increased T2/FLAIR signal intensity of right frontal lobe. Presurgical evaluation led to definition of epileptogenic network in a specific area of right frontal lobe. The resected specimen revealed MOGHE*. Discussion*. MOGHE appears to be a brain entity which shares some unique histopathological features. Review of the literature is in accordance with our patient's findings. The major neuropathological finding consists of areas with blurred gray-white matter boundaries due to heterotopic neurons in white matter and increased numbers of subcortical oligodendroglial cells with increased proliferation. MR abnormalities are present in T2/FLAIR sequences. It concerns patients with refractory frontal lobe epilepsy and appears to associate with unfavourable postsurgical outcome in seizure control.

**Conclusion:**

More cases are needed in order to establish more data about this distinct entity in frontal lobe epilepsy. This could be valuable knowledge to patients and doctors concerning expectations or management of undesirable outcome in frontal lobe epilepsy surgery.

## 1. Introduction

According to the updated classification for malformations of cortical development, there are three main categories of malformations which include the majority of histopathological findings and characteristics [1]. Nevertheless, a percentage of 2-26% of epilepsy surgery specimens are histopathologically classified as nonlesional [2].

There is an developing interest in the international literature on MOGHE (Mild Malformation of Cortical Development with Oligodendroglial Hyperplasia and Epilepsy). It is a new histopathological entity of brain lesions which cannot be included within the existent classification. We report the case of an epileptic patient with refractory epilepsy, who underwent surgical resection and had MOGHE.

## 2. Case Presentation

A 33-year-old male patient was suffering from epileptic seizures since the age of sixteen. He is right handed and his personal and familial medical history were free. At 16, he had three episodes with bilateral tonic clonic seizures at awakening. After the beginning of medication he presented only with focal hyperkinetic seizures with mild impairment of awareness without secondary generalization.

Because of drug-resistance he presented for presurgical evaluation. His seizure frequency was 4-8 seizures per month. His medication was lacosamide 600 mg/day, levetiracetam 3000 mg/day, and valproic acid 1000 mg/day.

**Figure 1:**
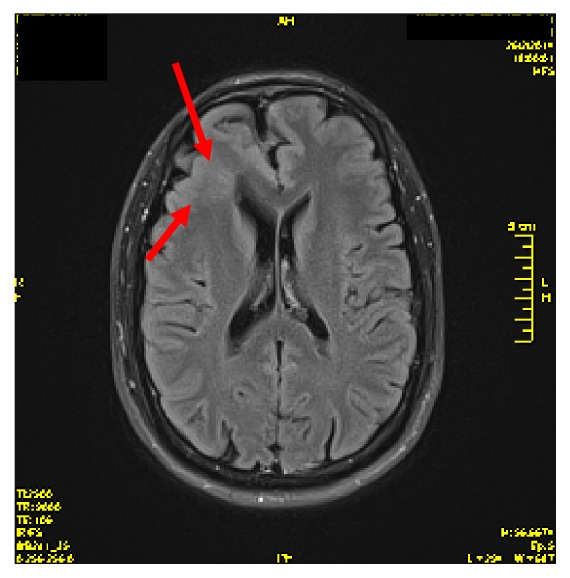
MRI-FLAIR sequence.

**Figure 2:**
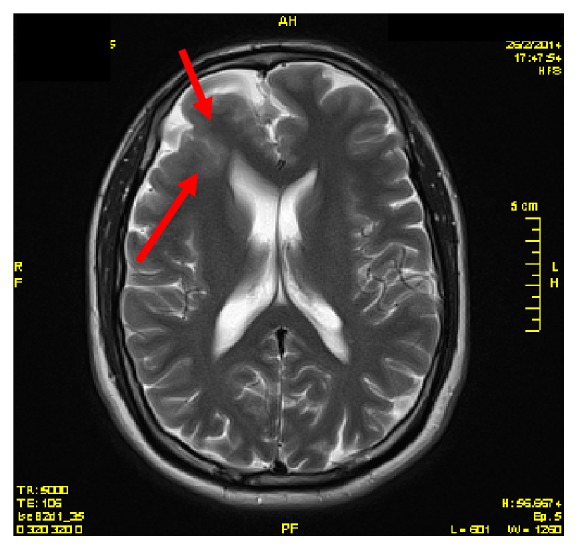
MRI-T2 sequence.

Brain MRI revealed increased T2 and FLAIR signal intensity of right frontal lobe, due to possible gray matter heterotopias or FCD (1.5 Tesla MR imaging study), Figures 1 and 2.

The patient underwent long term video-EEG with surface electrodes for three days, where five seizures were recorded with variable duration from 16 to 30 seconds.

Data suggested that epileptogenic network was at right frontal lobe. We proceeded thus with implantation of subdural grids and strips and depth electrodes to this specific area, in order to achieve a precise localization of caudal boundaries of epileptic network as well as to map the cortical functions, especially the kinetic area.

Second long term video-EEG and subsequent cortical stimulation led to a tailored resection of the right frontal lobe with respect to the kinetic area.

The available postoperative data concern a period of two years of seizure freedom.

**Figure 3:**
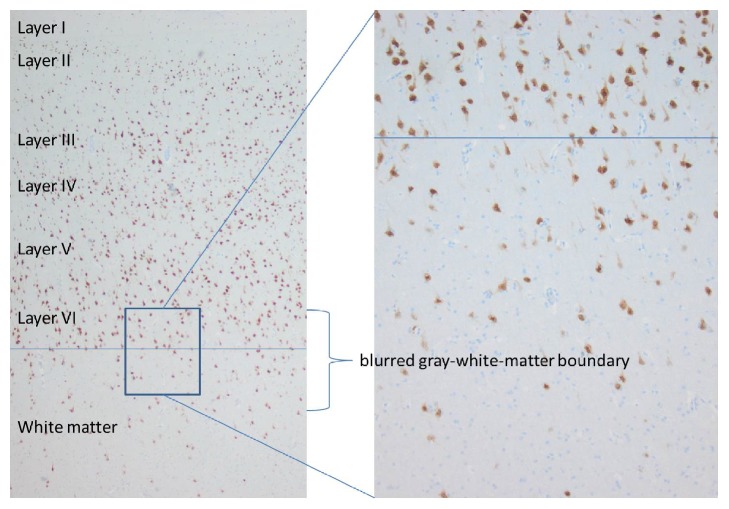
NeuN immunohistochemistry (neurons are labeled).

**Figure 4:**
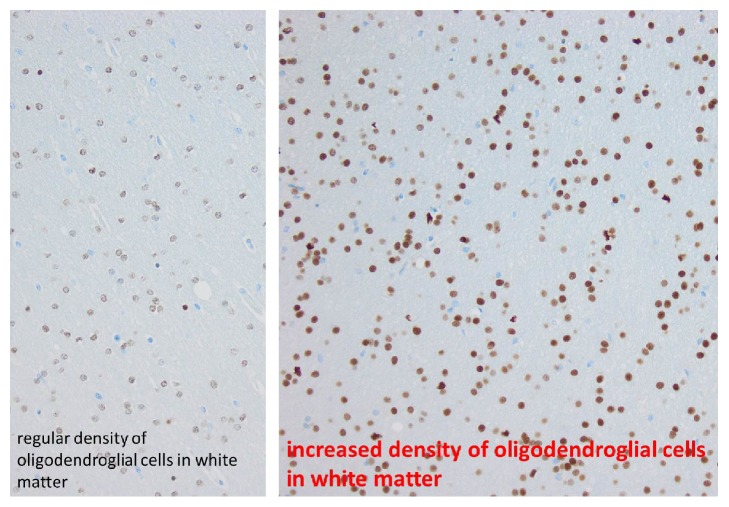
Olig2 immunohistochemistry (oligodendroglial cells are labeled).

**Figure 5:**
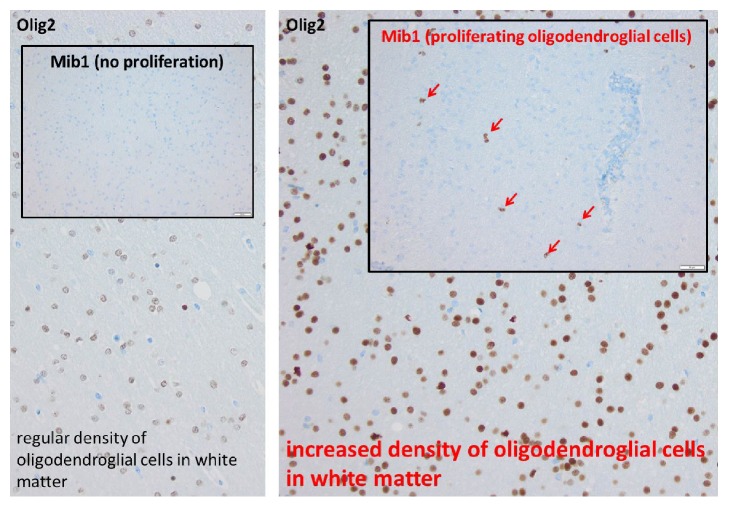
MIB1 (Ki67) immunohistochemistry (proliferating cells are labeled).

The histopathological findings of the resected brain specimen revealed the emerging entity called MOGHE, Figures 3, 4, and 5.

## 3. Discussion 

MOGHE's major neuropathological finding consists of areas with blurred gray-white matter boundaries due to heterotopic neurons in white matter and increased numbers of subcortical oligodendroglial cells [2]. Interestingly, these foci of heterotopic neurons are observed in the adjacent white matter and not in deep areas of white matter, defined as 500 *μ*m distant from the cortical junction.

The absence of heterotopic neurons in deep white matter excludes its histopathological classification as mild malformation of cortical development (MCD) Palmini Type II [3].

Another characteristic feature is the increase of Olig2-immunoreactive oligodendroglia observed in white matter and deep cortical layers as well as increased proliferation. This increase is multifocal with patchy areas of diffuse infiltration, which differentiates it from observed perivascular clustering in specimens of temporal lobe epilepsy [4].

Furthermore, in MOGHE there is no evidence of dysmorphic neurons nor balloon cells.

There are also no signs for vertical nor a horizontal dyslamination. Consequently, it cannot be classified within the ILAE classification scheme for FCD Type I or II.

Likewise, the histopathological report for our patient's resected specimen is presented with all the above features in Figures 3, 4, and 5.

Schurr* et al.* presented a series of 1381 en bloc resected epilepsy surgery brain specimens from which 3.7% could not be histopathologically classified and were considered nonlesional [2]. In 22 of these patients (42%) the above-mentioned histopathological findings of MOGHE were present.

All patients suffered from frontal lobe epilepsy, as our patient.

Clustered oligodendroglial hyperplasia with increased proliferative activity in white matter involving the gray-white matter junction was a common finding in all specimens.

Proliferation activity of oligodendrocytes was age varied. Specifically, it was higher in young patients undergoing operations compared with older patients of their series.

Authors suggested either a maldevelopmental or secondary regenerative component in the pathogenesis of these lesions. In our specimen no signs of diffuse neuroepithelial tumor infiltration (in particular oligodendroglioma) were observed. Furthermore, IDH mutations were absent, and detection of chromosomal 1p/19q losses did not reveal any codeletion in the oligodendroglial cell component.

There is a wide histopathological spectrum of epilepsy-associated brain lesions, including malformation of cortical development (MCD) as well as focal cortical dysplasias (FCD) which are a localized forms of MCD [5].

Their classification is well established and includes FCD Type I (Ia, Ib, Ic), Type II (IIa, IIb), and Type III (IIIa, IIIb, IIIc, IIId) [6].

Palmini's classification proposed in 2004 was widely used [3] whereas in 2011 Blumcke et al. recommended a new system for classifying FCD, resulting in the first international consensus classification of FCD proposed by the International League against Epilepsy (ILAE) in 2011 [7].

Nevertheless, MOGHE cannot be classified within this sort as it presents with the above unique histopathological features.

Oligodendroglial hamartoma is another lesion that should also be under consideration. Marucci et al. [8] described a case report of this particular lesion situated in frontal lobe, associated with drug resistant epilepsy and composed exclusively of mature oligodendroglial cells. Despite the scarcity of this extratemporal location, as well as the unusual histopathological feature of exclusive mature oligodendroglial cells, their lack of proliferative activity would help the differential diagnosis.

In 2018, Till Hartlieb* et al.* presented a series of 40 preoperative MRIs of twenty-five epileptic patients with histologically confirmed MOGHE in the resected brain specimens [9]. Mean age was 9.3 years old (pediatric population) and median age at MRI imaging was 5.2 years. They resulted in 2 age-related MR subtypes.

Subtype I consists of increased T2 and fluid attenuated inversion recovery (FLAIR) signal at the corticomedullary junction and occurred at a median of 2.6 years. Subtype II consists of reduced corticomedullary differentiation because of increased signal of the adjacent white matter and occurred at a median of 14.1 years.

The authors concluded that patchy areas of hypomyelination in histology seem to disappear during brain maturation and may therefore represent the histologic correlation of laminar T2 and FLAIR hyperintensities in subtype I. Furthermore, an age-related conversion of subtypes can be observed between 3 and 5 years.

Reviewing the literature on this field, we also found a reference of MR imaging identification of oligodendroglial hyperplasia by Hamilton* et al.* The authors presented a case of epileptic patient with refractory epilepsy who underwent a presurgical evaluation [10]. Findings of 1.5T MR imaging study revealed no abnormality, whereas subsequent 3T findings revealed abnormalities corresponding to the right frontal lobe electrophysiologic focus. Specific MR protocol for epilepsy evaluation was used and revealed subtle cortical thickening and blurring of the gray-white matter junction in the right frontal lobe compared to the residual normal cortex. Histologically, the resected specimen demonstrated oligodendroglial hyperplasia, which is characterized by the presence of increased number of normal-appearing oligodendroglial cells in the cortex and juxtacortical white matter. Oligodendroglial hyperplasia is considered to be at the mildest end of the spectrum of cortical migrational disorders [10]. It is a common characteristic with MOGHE and they share analogous MR findings.

Schurr* et al.* presented MR findings of MOGHE patients in their series, demonstrating the following features: frontal lobe localization, involvement of gray-white matter boundaries, increased signal intensity in FLAIR protocols, and presence of multifocal lesions. The authors suggested that the latter indicates a large anatomical extent of MOGHE, so this could account for surgical failure due to subtotal resections in their patient series. More specifically, postoperative seizure evaluation was available in 18 of 22 MOGHE patients. 33% were free of disabling seizures, whereas the majority (61%) of patients presented with ongoing seizures. One patient had rare disabling seizures, and two patients were reoperated due to seizure relapse. Second resection revealed histopathological remnants of MOGHE in both patients. This indicates that MOGHE could be associated with unfavourable outcome in surgery of frontal lobe in drug resistant epilepsy patients [2]. This does not apply to our patient so far, as the available data indicate two years of seizure freedom.

## 4. Conclusion 

MOGHE seems to be an emerging histopathological entity of brain lesions which shares some unique characteristics. These features, in combination with the absence of other characteristics of well-established malformations of cortical development, raise the difficulty of classifying this entity within the existent classification scheme.

Findings of our case report are in accordance with the provided data of the reviewed literature on this field.

The potential unfavourable outcome in seizure control could unequivocally be valuable knowledge to patients along with doctors. It concerns expectations for seizure freedom as well as management of undesirable outcome.

More cases of epileptic patients are definitely needed in order to establish more data about this distinct entity in frontal lobe epilepsy.

## Abbreviations

MRI: Magnetic resonance imaging
FLAIR: Fluid attenuated inversion recovery
IDH mutations: Mutations in isocitrate dehydrogenase
EEG: Electroencephalogram.
